# Massive extrapancreatic solid pseudopapillary neoplasm misdiagnosed as hepatic tumor: a case report and literature review

**DOI:** 10.3389/fonc.2024.1342400

**Published:** 2024-02-06

**Authors:** Jixu Guo, Qingjuan Zhao, Liting Qin, Shengjie Xie, Shiliu Lu, Baibei Li, Meilin He, Linhong Xie, Shuiping Yu

**Affiliations:** ^1^ Division of Hepatobiliary Surgery, The First Affiliated Hospital of Guangxi Medical University, Nanning, China; ^2^ The First Clinical Medical College, The First Affiliated Hospital of Guangxi Medical University, Nanning, China; ^3^ Division of Pathology, The First Affiliated Hospital of Guangxi Medical University, Nanning, China; ^4^ Division of Hepatobiliary Surgery, The Second People’s Hospital of Qinzhou, Qinzhou, China

**Keywords:** solid pseudopapillary neoplasms, retroperitoneum, local invasion, misdiagnosis, case report

## Abstract

**Background:**

Solid pseudopapillary neoplasms (SPNs) of the pancreas are uncommon, low-malignancy neoplasms. Moreover, the occurrence of extrapancreatic SPNs is rarely encountered.

**Case summary:**

A 45-year-old female presented with a right upper abdominal mass and abdominal pain for 3 and 1 months as chief complaints, respectively. Initially, the patient was misdiagnosed with hepatocellular carcinoma based on her symptoms and results of physical and imaging examinations. Following multidisciplinary discussion and ruling out surgical contraindications, a decision was taken to proceed with surgical intervention. Interestingly, the tumor was found to originate from the retroperitoneum and had invaded the right half of the liver and the right wall of the inferior vena cava. The operation was uneventful, and the pathological findings confirmed the tumor as an extrapancreatic SPN. The patient remained asymptomatic after 15 months of follow-up.

**Conclusion:**

Surgical treatment remains the preferred option for extrapancreatic SPN. The preoperative misdiagnosis also highlights the importance of accurate diagnosis and the development of appropriate treatment strategies for liver masses.

## Introduction

Solid pseudopapillary neoplasms (SPNs) of the pancreas, initially described by Frantz in 1959, are rare neoplasms with low-malignant potential neoplasm (1). Surgical treatment remains the gold standard for the treatment of SPNs, and this disease is generally associated with a favorable prognosis if diagnosed and treated early (2–6). In addition, SPNs are also detected in other parts of the body, called extrapancreatic SPNs (7). To date, extrapancreatic SPNs have only been reported as sporadic cases in the literature, with poorly understood research characteristics. Here, we presented a case of an extraperitoneal SPN with local invasion, which may be the largest and the only documented case of retroperitoneal extrapancreatic SPN with local invasion. Meanwhile, recently encountered cases of extrapancreatic SPN were also reviewed to enhance our understanding and management of these rare tumors.

## Case presentation

### Chief complaints

A 45-year-old female was admitted to the hospital in August 2022 due to the presence of a right upper abdominal mass and pain.

### History of present illness

The patient presented with a mass in the right upper abdomen three months ago, accompanied by recurrent abdominal pain. She underwent various examinations, including a CT scan and ultrasound, at the local hospital. According to the test results, the diagnosis of hepatocellular carcinoma was made. However, the liver biopsy revealed a neuroendocrine tumor of grade 2 (G2), which contradicted the previous findings. Thereafter, immunohistochemical analysis was positive for Syn, CD56, and Vimentin markers in the tumor cells, whereas markers such as CK7, CK9, CK20, Arg-1, CDX-2, and NSE yielded negative results. In order to obtain a more accurate diagnosis and determine the appropriate treatment course, the patient was admitted to our hospital.

### History of past illness

No significant past medical history, especially liver disease, such as hepatitis, alcoholic or non-alcoholic liver disease, or cirrhosis.

### Personal and family history

The patient had no family history or genetic predisposition for the disease.

### Physical examination

An abdominal mass was detected 7 cm below the costal line at the right mid-clavicular and 10 cm below the xiphoid process. The mass exhibited a hard, smooth-surfaced, firm texture with unclear boundaries. Lastly, no evidence of jaundice, splenomegaly, or lymph node enlargement was observed.

### Laboratory examinations

The routine results, including bloodwork, coagulation, and liver function tests, were within the normal range. It is worthwhile noting that the patient tested negative for hepatitis B or C. Additionally, the patient’s Protein Induced by Vitamin K Absence or Antagonist II (PIVKA-II) level was 50.38 mAU/ml (reference: 0-40 mAU/ml), whereas the levels of carcinoembryonic antigen (CEA), cancer antigen (CA)-125, CA-199 and alpha-fetoprotein (AFP) were normal.

### Imaging examinations

**Figure 1:**
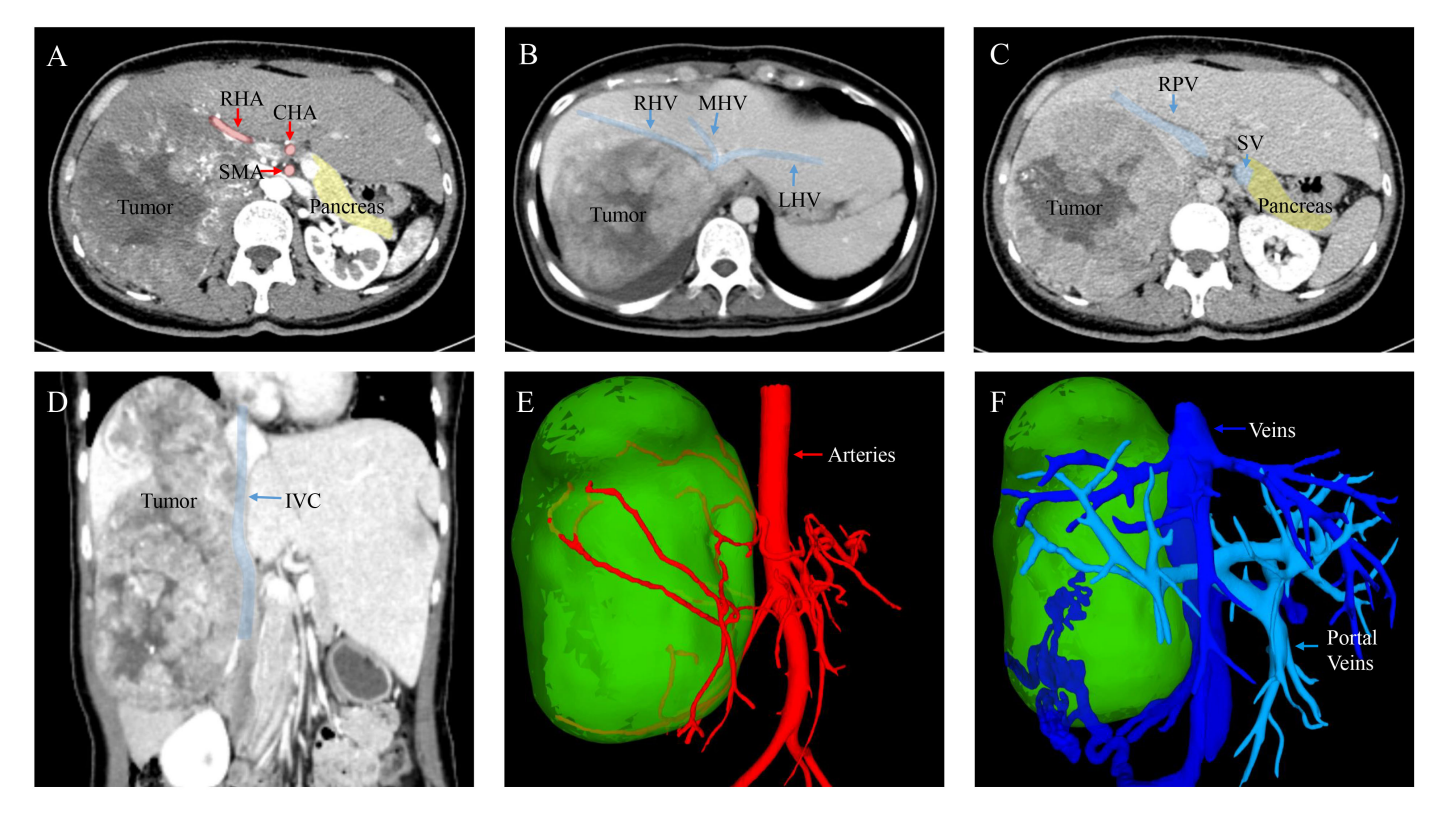
Images of contrast-enhanced CT scan and Three-dimensional reconstruction. **(A)** Arterial phase. **(B)** Portal venous phase. **(C)** Delayed phase. **(D)** Coronal plane. During the arterial phase of contrast enhancement, the mass illustrating rapid and pronounced enhancement. However, in the subsequent portal venous and delayed phases, the enhancement steadily diminishes. **(E, F)** Three-dimensional reconstruction based on contrast-enhanced tomography (CT) depicting the relationship between the tumor and arteries, as well as veins. RHA, Right Hepatic Artery; CHA, Common Hepatic Artery; SMA, Superior Mesenteric Artery; RHV, Right Hepatic Vein; MHV, Middle Hepatic Vein; LHV, Left Hepatic Vein; RPV, Right Portal Vein; SV, Splenic Vein; IVC, Inferior Vena Cava.

Based on the findings of contrast-enhanced ultrasound, the mass is likely to be malignant. Afterward, the CT scan displayed a low-density hepatic mass (**Figure 1A**). The mass showed uneven enhancement with thickened and tortuous tumor vessels in the arterial phase (**Figure 1B**). In contrast, the enhancement was lower in the portal venous and delayed phases (**Figures 1C, D**). Patchy filling defects were visible in the right hepatic vein. No abnormality was observed in the remaining liver, bile ducts, gallbladder, pancreas, or lymph nodes. Based on the CT findings, a preliminary diagnosis of right lobe giant hepatocellular carcinoma with a thrombus in the right hepatic vein was considered. Three-dimensional reconstruction based on contrast-enhanced tomography revealed the relationship between the tumor and intrahepatic bile duct, portal vein, hepatic artery, and inferior vena cava (**Figures 1E, F**). Furthermore, the patient’s indocyanine green retention rate at 15 minutes (ICGR15) is less than 10%. According to the formulae of Urata et al. (8), the patient’s standard liver volume was calculated to be 1031.53 cm3, and the liver volume after right hemihepatectomy was 649.34 cm3, accounting for 62.94% of the standard liver volume.

## Final diagnosis

The preoperative primary diagnosis was hepatocellular carcinoma, while the diagnosis of neuroendocrine tumor was not excluded. However, postoperative pathological examination confirmed that the tumor was an extrapancreatic solid pseudopapillary neoplasm, indicating a preoperative misdiagnosis.

## Treatment

**Figure 2:**
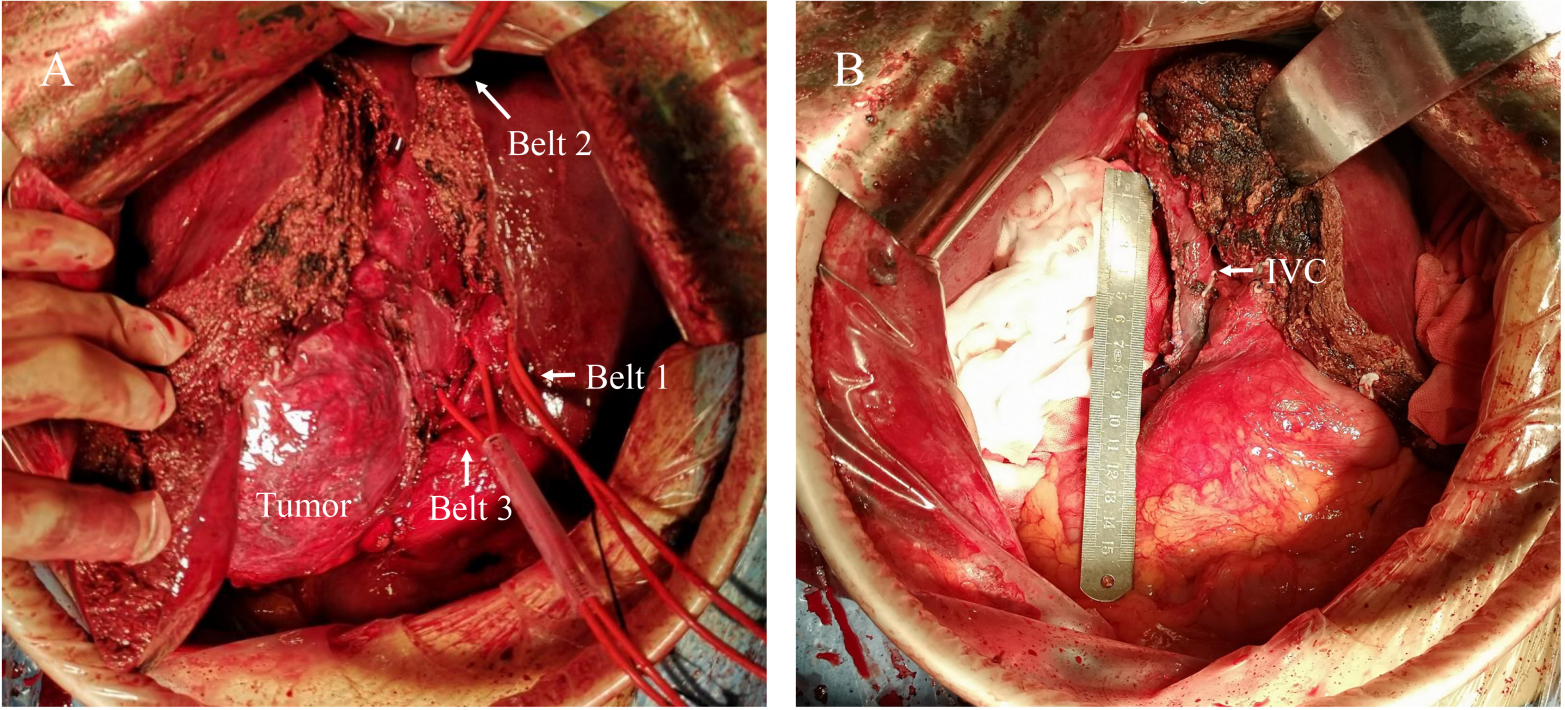
Intraoperative findings and surgical procedure. **(A)** The tumor was mainly located in the right subhepatic space with local invasion. The belt 1 represents the Pringle maneuver used to control hepatic portal blood flow. Belt 2 was applied to the suprahepatic inferior vena cava and belt 3 to the subrenal inferior vena cava for complete hepatic vascular occlusion. **(B)** After the tumor was completely excised, the inferior vena cava was repaired. IVC, Inferior Vena Cava.

Despite the primary diagnosis strongly suggestive of stage C or stage IIIa hepatocellular carcinoma according to the Barcelona Clinical Liver Cancer (BCLC) staging system (9) or the China Liver Cancer (CNLC) staging system (10), respectively, the diagnosis of neuroendocrine tumor from a biopsy performed at another hospital prompted us to proceed with surgery. The tumor was approximately 20 cm×20 cm in size and was predominantly situated in the right subhepatic space, with a complete capsule and a clear boundary. Meanwhile, ascribed to its large size, the tumor obstructed the right renal vein and inferior vena cava, causing compensatory proliferation and compression of retroperitoneal veins. The tumor had also invaded the right hepatic lobe as well as the right wall of the inferior vena cava. All abdominal lymph nodes appeared to be normal. Based on our intraoperative findings, we concluded that the tumor likely originated from the retroperitoneum. Therefore, the patient underwent a surgical procedure for the treatment of a retroperitoneal tumor involving the inferior vena cava and the right liver lobe. The first hepatic hilum was meticulously dissected, followed by the ligation and division of the right hepatic artery and the right branch of the portal vein. A demarcation line was identified, and the liver was divided into left and right lobes along the ischemic demarcation line. Next, the right hepatic vein was meticulously dissected and ligated. The tumor in the retroperitoneal space was fully mobilized and exposed. The suprahepatic and infrarenal inferior vena cava were then clamped with a prepositioned vascular occlusion belt to achieve complete hepatic vascular occlusion (**Figure 2A**). Subsequently, the tumor involving the right lobe of the liver infiltrating the wall of the inferior vena cava and the entire affected segment of the inferior vena cava was resected. The inferior vena cava was meticulously repaired using 5-0 prolene sutures to restore continuity after tumor resection (**Figure 2B**). The patient underwent a 300-minute surgical procedure with an estimated blood loss of 1000 mL and 4 units of red blood cells transfused intraoperatively. The patient made a satisfactory recovery postoperatively with no complications and was discharged 10 days postoperatively.

## Pathology

**Figure 3:**
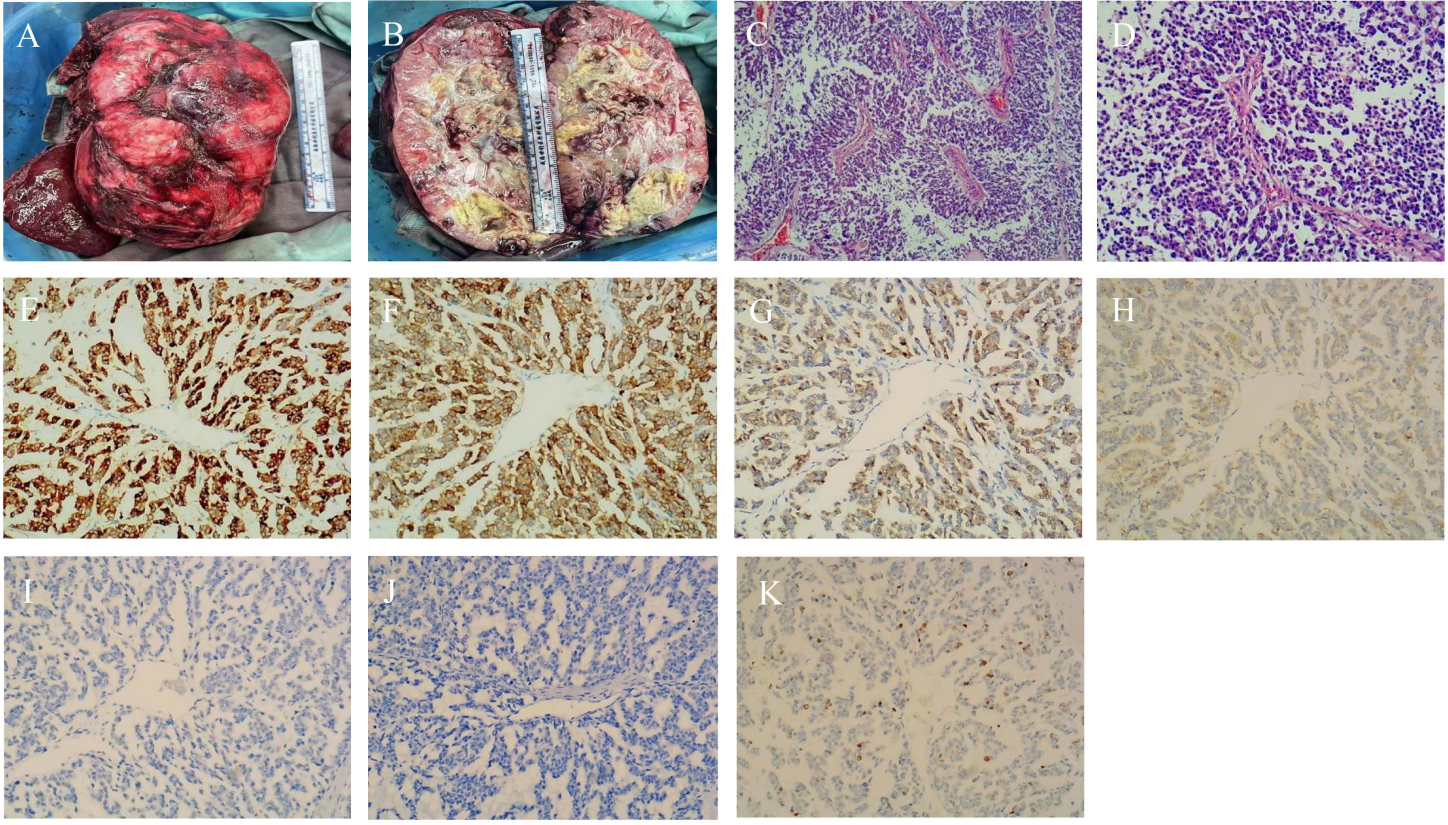
Pathological and Immunohistochemical images. **(A, B)** Microscopic view of the tumor; **(C)** Tumor cells aggregate around fibrous blood vessels, forming a pseudopapillary structure. (×40); **(D)** Nuclei are round or oval with longitudinal grooves, resembling coffee beans. (×100); **(E–G)** Immunohistochemical staining revealing CD10-, CD56-, and β-catenin-positivivty (×100); **(H)** Immunohistochemical assay exposing partial Syn positivity (×100); **(I)** Immunohistochemical staining portraying CgA negativity. (×100); **(J)** Immunohistochemical staining portraying CD99 negativity. (×100); **(K)** Positive staning for Ki-67 in tumor cells (×100).

Postoperative pathology confirmed that the tumor was an extrapancreatic solid pseudopapillary neoplasm. An intact encapsulated mass measuring 19×12×10 cm was visible, with a gray-white, gray-yellow, and gray-red solid cut surface and a clear demarcation from the surrounding tissue (**Figures 3A, B**). Microscopically, the tumor was composed of solid and pseudopapillary structures. In the solid region, tumor cells were characterized by poor adhesion and abundant small blood vessels. On the other hand, pseudopapillary structures were formed when tumor cells located farther from fibrovascular cords degenerated. The morphology of the tumor cells was relatively consistent, with round or oval nuclei, longitudinal nuclear grooves, and irregular nuclear membranes resembling coffee beans. The tumor was well-demarcated but focally infiltrated the adjacent liver tissue. Besides, a vascular tumor thrombus was present, but there was no evidence of nerve invasion. To confirm the diagnosis and differential diagnosis, immunohistochemistry staining was performed. As anticipated, the tumor cells exhibited nuclear and cytoplasmic expression of β-catenin. The tumor cells were positive for CD10, Vimentin, CD56, partially positive for Syn, and negative for CgA, Arginase-1, Hepatocyte, Glypican-3, CK19, CD99, and CK7. Finally, the Ki-67 staining index was 15% (**Figures 3C–K**). Based on morphology and immunohistochemistry, hepatocellular carcinoma and cholangiocarcinoma were easily ruled out, and further identification was needed between solid pseudopapillary neoplasm and neuroendocrine tumors. Considering nuclear and cytoplasmic expression of β-catenin and absent labeling for CgA, the extrapancreatic solid pseudopapillary neoplasm was diagnosed finally.

## Outcome and follow-up

The patient underwent regular follow-up assessments, and at the 15-month postoperative mark (November 20, 2023), the patient’s recovery was found to be favorable, accompanied by a satisfactory quality of life. The CT scans revealed no evidence of tumor recurrence within the retroperitoneal region, and the pancreas appeared normal. Lastly, the patient’s PIVKA-II level was within the normal range.

## Discussion

Solid pseudopapillary neoplasm of the pancreas (SPNP), as a rare low-malignant neoplasm, accounts for approximately 2% of all pancreatic neoplasms (7, 11). The disease has a female predilection, and the male/female ratio of incidence rate varies among different races (2, 3, 12, 13). The presentation of SPNP is related to its size (14). In the early stage, patients are usually asymptomatic owing to the small size of the tumor and the lack of endocrine function. With the growth of the tumor, the symptoms of tumor compression of the alimentary tract progressively appear, including abdominal pain, nausea, jaundice, and even intestinal obstruction and pancreatitis (3, 15–17). Noteworthily, the majority of SPNPs have been described as benign in nature; nonetheless, 10-15% of SPNPs exhibit malignant behavior and metastases (18). Surgery remains the current preferred treatment option for SPNP (2–4, 6, 19), and the surgical approach largely depends on the location, size, and nature of the tumor. Commonly employed surgical procedures include distal pancreatectomy, central pancreatectomy, total pancreatectomy, pancreaticoduodenectomy, etc. The prognosis of SPNP is generally favorable, with an overall 5-year survival rate of approximately 95% for patients who undergo surgical resection (20, 21). The postoperative recurrence rate of SPNP is 2%. Established risk factors for recurrence include male gender, positive lymph nodes, and R1 margins. Given the potential risk factors for recurrence, extended follow-up is crucial for patients, aiming to effectively monitor and manage potential recurrencess (22).

However, the incidence of extrapancreatic SPNs is extremely low, and all reported cases are sporadic. In 2022, Liu’s team reported a total of 50 cases of extrapancreatic SPNs since 1990 (7). Building on this, we attempted to research and summarize cases of extrapancreatic SPNs of primary retroperitoneum origin, including data up to November 2023, in **Supplementary Table 1** (23–27). For a comprehensive overview, all cases of extrapancreatic SPNs were included in **Supplementary Table 2**. The results inferred that extrapancreatic SPNs predominantly occur in females, with a male-to-female ratio of 68.62%. In addition, the most common locations for extrapancreatic SPNs, in descending order, are the ovary, testicular/paraperitoneal region, mesentery, and retroperitoneum. Additionally, Our study identified a total of 6 cases of retroperitoneal SPN, all occurring in females with a mean age of 34 years (range 22-47). Importantly, their symptoms were similar to those with pancreatic SPNs; that is, they were either asymptomatic or experiencing abdominal discomfort. All patients underwent surgery and were followed up for 6-15 months after surgery. No tumor recurrence or death of the patient was observed, implying positive outcomes. The mean size of the tumors was 12.6 cm (range 6-19 cm), with one case of local invasion, while the remaining cases showed no signs of metastasis or heterotopic pancreas. To the best of our knowledge, the extrapancreatic SPN reported in this study is the largest and the sole case, thereby warranting further investigation.

Laboratory and imaging examinations for extrapancreatic SPNs are frequently inconclusive (28). Besides, routine laboratory tests such as complete blood count, liver function, and electrolytes, as well as tumor markers such as CEA, CA125, CA199, and AFP, do not yield specific diagnostic results (29). Concerning the imaging features of SPN, its ultrasonic features chiefly display cystic and solid components in addition to uneven internal echoes. As the most extensively utilized objective examination, CT can provide some clues about the nature, location, and invasiveness of the tumor. The typical CT appearance of SPNs is a large, unevenly dense mass with solid and cystic components, with the latter principally located at the edge of the mass and the former localized in the center, often with a complete fibrous capsule (30). Ascribed to differences in location and the lack of specificity in imaging studies, distinguishing extrapancreatic SPNs from other abdominal masses such as ovarian tumors (cystadenomas or borderline tumors), retroperitoneal tumors (leiomyomas, lipomatous tumors, or gastrointestinal stromal tumors), and pelvic tumors (teratomas or cystadenoma-like lesions), is challenging.

Regarding the treatment of extrapancreatic SPNs, surgical treatment remains the preferred option (13). Due to the limited number of reported cases and the diverse locations of the tumors, surgeons had to adapt their surgical approaches accordingly. However, our case was unique in the sense that the condition was initially misdiagnosed as hepatocellular carcinoma and classified as stage IIIa hepatocellular carcinoma according to the CNLC staging system (10). We recommend CNLC-IIIa patients undergo thorough evaluations to determine the ideal treatment approach, including transarterial chemoembolization (TACE), tyrosine kinase inhibitor, or surgery. Indeed, delaying treatment decisions could result in missed opportunities for the most effective interventions. Additionally, based on the BCLC staging system (9), the patient would not be considered eligible for surgery. Furthermore, the survival rate of CNLC-IIIa or BCLC-C stages is relatively low. Fortunately, the results of the liver biopsy, which was indicative of a neuroendocrine tumor, prompted us to perform surgery, and the postoperative pathological examination indicated that the mass was an extraancreatic SPN, signifying that the patient may have a relatively positive prognosis. Taken together, this case showcases the importance of accurate diagnosis and appropriate treatment selection tailored to the patient’s actual medical condition. It also emphasizes the need for ongoing research and investigation into the diagnosis and treatment of liver masses to improve patient outcomes.

**Figure 4:**
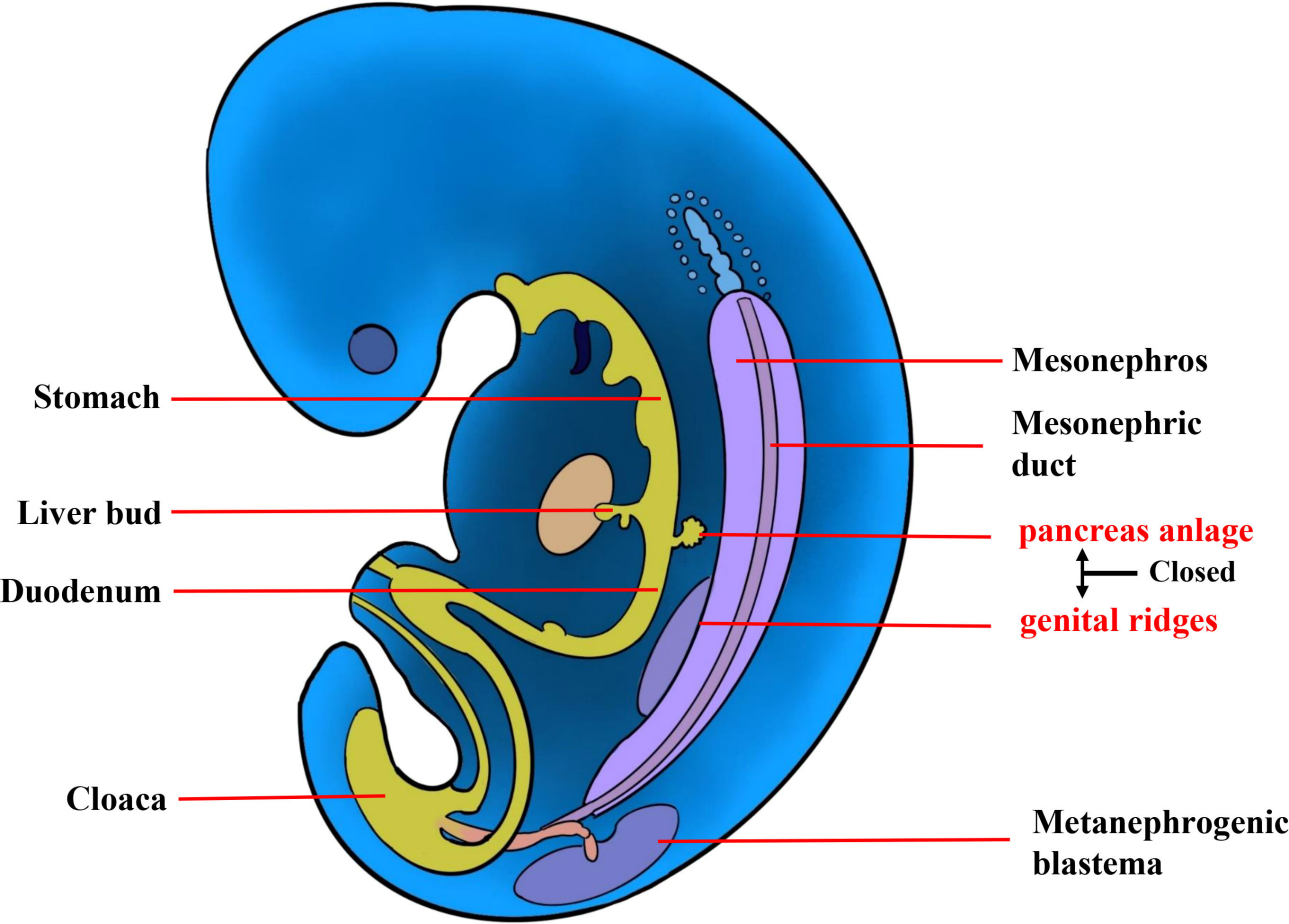
The etiology of extrapancreatic SPN. During early embryonic development, the genital ridge and primary pancreatic buds are in close proximity, and cells from the genital ridge may migrate to the pancreas.

The etiology of extrapancreatic SPNs remains elusive, and there are currently two mainstream hypotheses. The first is that extrapancreatic SPN is caused by an ectopic pancreas (31). Nevertheless, it cannot account for cases of extrapancreatic SPNs without evidence of ectopic pancreas, including the present case. The other hypothesis was proposed by Kosmahl et al. (32), suggesting that during early embryonic development, the genital ridge and primary pancreatic buds are in close proximity, and cells from the genital ridge may migrate into the pancreas (**Figure 4**). This theory may offer an explanation for extrapancreatic SPN cases without ectopic pancreatic tissue. However, its etiological mechanism warrants further investigation. In our case, the origin of the tumor from the retroperitoneum is only speculative based on our observations during surgery; however, the possibility of liver origin cannot be ruled out.

Pathology plays a paramount role in diagnosing SPN, particularly in extrapancreatic cases that are rare and often misdiagnosed. The resected tumor specimen was pathologically similar to SPNP macroscopically (33). The immunohistochemical results of the tumor cells revealed positivity for CD10, CD56, Vimentin, and partially for Syn. β-catenin expressed in the cytoplasm and nucleus of tumor cells. Notably, SPNs share similar tumor markers with other pancreatic tumors, such as neuroendocrine neoplasms (NENs) and pancreatic ductal carcinoma (PDCs), which led to a misdiagnosis in the patient’s liver biopsy at another hospital. However, cytoplasmic expression of β-catenin and absent labeling for CgA played a significant role in confirming the diagnosis. Furthermore, the absence of CD99 expression in the immunohistochemistry analysis assisted in distinguishing extrapancreatic SPNs from primitive neuroectodermal tumors (PNET). Therefore, the incorporation of additional immunohistochemical markers holds paramount significance in facilitating the diagnosis and differential diagnosis of SPNs. At the same time, recent studies have evinced that more than 90% of patients with SPNs have point mutations in the CTNNB 1 gene exon 3 of β-catenin (34). The CTNNB 1 gene is involved in the Wnt/β-catenin signaling pathway, and its point mutation prevents cytoplasmic β-catenin phosphorylation. Furthermore, it translocates to the nucleus, activates the Wnt/β-catenin signaling pathway and cyclin D1 genes, and induces nuclear overexpression of cyclin D1. The identification of nuclear translocation and accumulation of β-catenin protein in tumor cells provides an important pathological basis for the diagnosis of SPN. The Ki-67 protein is a widely recognized proliferative marker for human tumor cells, enabling the prediction of patient outcomes regarding metastasis and progression. Kang’s and Park’s research teams have presented compelling evidence that Ki-67 does not correlate with the malignant potential of SPN (35, 36). Nevertheless, in this particular case, a Ki-67 staining index of 15% (≥ 4%), along with intraoperative invasion, indicated that the patient was at a high risk of recurrence or metastasis post-surgery. Therefore, a postoperative review at two years is warranted.

## Conclusion

It is indeed a rare and interesting case, and the initial misdiagnosis of hepatocellular carcinoma accentuates the importance of accurate diagnosis and appropriate treatment planning. The discovery of local invasion, in this case, was also noteworthy, as it underscores the need for a thorough preoperative evaluation and intraoperative assessment to ensure complete resection of the tumor. The successful outcome of the surgery and the patient’s asymptomatic status following 15 months of follow-up is encouraging and suggests a good prognosis for extrapancreatic SPN when diagnosed and treated early. Overall, this case review analyzed the clinical characteristics and pathological hallmarks of SPNs in order to better understand this rare neoplasm and develop more effective diagnostic and treatment strategies in the future.

## Data availability statement

The original contributions presented in the study are included in the article/**Supplementary Material**. Further inquiries can be directed to the corresponding author.

## Ethics statement

Written informed consent was obtained from the individual(s) for the publication of any potentially identifiable images or data included in this article.

## Author contributions

JG: Writing – original draft. QZ: Writing – original draft. LQ: Writing – review & editing. SX: Writing – original draft. SL: Writing – original draft. BL: Writing – original draft. MH: Writing – original draft. LX: Writing – review & editing. SY: Funding acquisition, Writing – review & editing.
